# 

**Published:** 1922-06

**Authors:** 


### ANNOUNCEMENTS

National Association of Dental Examiners, will held their annual meeting in Los Angeles, July 17 and 18th.

National Association of Dental Faculties, will hold their annual meeting in Los Angeles, July 13, 14, 15, 1922.

National Society of Dental Prosthetists, will hold their meeting in Los Angeles, July 13 to 15.

American Dental Library Association, will hold its meeting July 17, in Los Angeles.

Xi Ssi Phi Alumni Fraternity, will hold its annual convention in Los Angeles, July 17th.

### NATIONAL DENTAL ASSOCIATION.

The annual convention of the National Dental Association is one the greatest and most potent channels through which dental knowledge comes to us.

Attendance at these meetings gives the inspiration and desire to "do dentistry that will add ten years to the average of human life."

Los Angeles, California, invites you to participate in one of the best conventions of the National Dental Association ever held.

Hotel Ambassador is the place.

July 17-21, 1922, the time, and you are advised to make railway and hotel reservations now. The Publicity Committee of the N. D. A., Bert Boyd, Chairman.

### DELTA SIGMA DELTA FRATERNITY.

The Thirty-eighth Annual Meeting of the Supreme Chapter of Delta Sigma Delta Fraternity will be held in the Alexandria Hotel, Los Angeles, Cal., on Monday, July 17, 1922, at 9:30 A. M.

The regular order of business will be carried out both at the morning and afternoon sessions until finished, to be followed by the initiatory exercises in the afternoon. All of our activities will be held in the above-mentioned hotel, including the luncheon at noon immediately after the morning session, and the banquet in the evening. A rest room and registration booth have also been provided for our members in the Ambassador Hotel, the headquarters of the National Dental Association.

Those expecting to attend should promptly notify Dr. Frederick M. Hunt, 709 Walter P. Story Building, Los Angeles, Cal.

By order of the Supreme Chapter, CHALMERS J. LYONS, Supreme Grand Master; R. HAMILL D. SWING, Supreme Scribe.

---

### PACIFIC COAST SOCIETY OF ORTHODONTISTS

A cordial invitation is extended to all interested in orthodontia to attend the next annual meeting of the Pacific Coast Society of Orthodontists, which will be held in Los Angeles, California, July 13, 14 and 15, 1922. Those who contemplate being in attendance are requested to make known their intention to the Secretary as soon as possible. CARL O. EEGSTROM, *Secy.-Treas.*, Box 1070, Sacramento, Cal.

---

### MEETING OF THE NATIONAL ALUMNI CHAPTER OF THE PSI OMEGA FRATERNITY

The National Alumni Chapter of the Psi Omega Fraternity will hold its next meeting on Monday, July seventeenth, 1922, at the Clark Hotel, Los Angeles, California. The Chapter will be called to order at ten A. M. There will also be an afternoon meeting and an informal banquet at eight P. M. at the Los Angeles Athletic Club, at which the Psi Omega Ladies will be the guests of the Fraternity. A large attendance is desired. Every Psi Omega should register immediately on arrival in Los Angeles. Headquarters will be maintained during the National Meeting at both the Ambassador and Clark Hotels. MAX WASSMAN, JR., *Grand Master*.

---

### DR. WILLIAM VAN BERGEN AMES.

Dr. Ames died at his winter home in Arizona, February 23rd, 1922. He was born at Attica, Ohio, May 27th, 1859.

His father was an eminent physician and the family later moved to Fremont, Ohio, where the son attended public school until he entered Notre Dame at South Bend, Indiana. He entered Ohio Dental College at about twenty years of age, graduating in the spring of 1880. During his college course he won a number of medals offered by the faculty for the best work in the different branches taught. He won gold medals for prosthetic work and for superior work in chemistry.

Dr. Ames located in Chicago, Illinois, in 1881 and soon came into prominence as a dentist of special ability, making improvements in denture construction and prosthetic appliances in general. He was one of the original inventors of the gold inlay. With four others he organized the Northwestern University Dental School in 1891 and was for several years Professor of Prosthetic Dentistry in that institution.

Aside from his original work in dentistry he devoted much time to originating dental cements, but did not push this investigation until it became evident that his eyes were failing for close dental work. When satisfied that he had originated cement that would be of real value to the profession and the public, he placed the outcome of his original chemical work on the market. And Ames cement is now known throughout the world.

In 1895 Dr. Ames married Miss Virginia Gilmer, daughter of Dr. T. L. Gilmer, Chicago, and their married life was a happy one. In 1913 he bought a farm of eighty acres near Lake Forest, Ill., and in the summer of 1914 had his first taste of living in the country. From time to time he added to the original tract and at the time he died the farm had increased to eight hundred acres. The war and the plea to farmers to farm for our Allies and for the United States caused him to farm to the limit of his land. Farming under war conditions was a difficult task for any tried farmer and contending with the many problems which arose seriously impaired Dr. Ames' health, and he loaned one hundred and ninety acres to the Women's Land Army of America and this tract was cultivated for war purposes by farmerettes.

For the past several years Dr. Ames has spent his winters in Arizona. In 1921 he built a large home in the desert near Chandler and it was here that he passed away.

Dr. Ames was a member of the National Dental Association, Illinois State, Chicago Dental Society and Odontological Society of Chicago. He was a genial man, beloved by all who knew him, and he will be missed by the dental profession.—*Dental Summary*, May.

